# Burden and Determinants of Drug–Drug Interactions at Hospital Discharge: Warfarin as a Model for High-Risk Medication Safety

**DOI:** 10.3390/clinpract16010008

**Published:** 2025-12-31

**Authors:** Kanthida Methaset, Arom Jedsadayanmata

**Affiliations:** 1 Division of Pharmacy Services, Thammasat University Hospital, Pathum Thani 12120, Thailand; kanthida@tu.ac.th; 2 Graduate Program in Drug Utilization and Health Outcomes Research, Faculty of Pharmacy, Thammasat University, Pathum Thani 12120, Thailand; 3 Faculty of Pharmacy, Thammasat University, Pathum Thani 12120, Thailand

**Keywords:** burden, determinant, drug interaction, hospital discharge, polypharmacy, warfarin

## Abstract

**Background**: Potential drug–drug interactions (pDDIs) present substantial challenges to medication safety during care transitions. Warfarin, with its narrow therapeutic index and extensive interaction profile, provides a strategic model for examining pDDIs at discharge. This study aimed to characterize the burden and determinants of major warfarin pDDIs among patients discharged from a tertiary-care hospital. **Methods**: This retrospective cross-sectional study analyzed electronic health records of 1667 patients discharged home on warfarin. Major pDDIs were identified using the Micromedex^®^ Drug Interaction database. Log-binomial regression was used to assess predictors of ≥1 major pDDIs, and generalized Poisson regression was used to model the number of pDDIs per patient. **Results**: Major warfarin pDDIs were identified in 81.6% (95% CI: 79.6–83.4%) of patients at hospital discharge. The burden was considerable: 35.1% (95% CI: 32.8–37.4%) of patients had one major pDDI, while 46.5% (95% CI: 44.1–48.9%) had two or more. Polypharmacy (≥5 concurrent medications) was the strongest predictor, associated with a higher risk of any major pDDI (adjusted risk ratio 1.72, 95% CI: 1.46–2.02) and nearly three times the burden of interactions per patient (adjusted incidence rate ratio (IRR) 2.87, 95% CI: 2.36–3.49). When modeled as a continuous variable, each additional discharge medication was associated with a 9% increase in predicted pDDI burden (IRR 1.09, 95% CI: 1.08–1.10). **Conclusions**: Using warfarin as a model for high-risk medication safety, major pDDIs were highly prevalent at hospital discharge, with polypharmacy as a significant predictor of both the presence and burden of interactions. These findings emphasize the importance of identifying polypharmacy-related pDDIs to reduce potential drug interaction risk during care transitions.

## 1. Introduction

Potential drug–drug interactions (pDDIs), particularly those involving high-risk medications, are a common cause of preventable harm and remain a primary patient-safety concern during transitions of care [1,2,3,4]. Transitions from hospital to home are widely recognized as periods of heightened vulnerability for medication-related harm, with medication discrepancies and communication gaps contributing substantially to preventable adverse drug events [5,6,7]. At hospital discharge, patients frequently receive new or modified medication regimens to manage multiple comorbidities at home, increasing the likelihood of pDDIs [8,9,10,11,12,13,14,15]. Despite their clinical importance, studies examining pDDIs at hospital discharge often do not focus specifically on high-risk medications [8,9,10,11,12,13,14,15]. Consequently, the overall burden and determinants of pDDIs involving high-risk medications at discharge are not well characterized.

Certain medications are especially susceptible to clinically meaningful pDDIs because of their narrow therapeutic index, extensive interaction profile, and require careful monitoring. Warfarin is one of the clearest examples. It interacts with many commonly used drugs that may alter the international normalized ratio (INR), and subtherapeutic or supratherapeutic INR levels are associated with major bleeding or thromboembolic events [16,17,18,19,20,21]. These risks can be amplified at hospital discharge, when interacting medications such as antibiotics, analgesics, and cardiovascular agents are frequently initiated, discontinued, or dose-adjusted, and when delays in post-discharge INR monitoring may further destabilize anticoagulation control. Warfarin remains the preferred treatment for specific patient populations, such as patients with mechanical heart valves and severe kidney disease [22,23]. In addition, warfarin continues to be widely used in low-resource healthcare settings across many low- and middle-income countries, including Thailand, because of its affordability and limited access to direct oral anticoagulants within public healthcare systems. Consequently, patients discharged on warfarin in these settings may face a substantial risk of pDDIs during care transitions. Yet, evidence characterizing this burden at hospital discharge remains limited outside high-income countries. Together, these characteristics make warfarin a valuable model for examining the burden and determinants of pDDIs involving high-risk medications at hospital discharge.

Despite warfarin being a well-recognized high-risk medication and a valuable model for studying pDDIs, much less is known about the overall burden of major warfarin pDDIs that patients encounter at discharge or the patient-level factors associated with these interactions. Prior research has typically examined isolated warfarin–drug interaction pairs, outpatient anticoagulation management, or adverse outcomes following exposure. However, few studies have systematically assessed the cumulative burden of warfarin pDDIs specifically at the point of discharge, or identified which patient characteristics predispose individuals to multiple major interactions during this transition. A more comprehensive understanding of this burden is needed to inform safer prescribing practices and targeted interventions during care transitions. Therefore, using warfarin as a model high-risk medication, the present study aimed to (1) quantify the prevalence and patient-level burden of major warfarin pDDIs at hospital discharge and (2) identify factors associated with both the presence and number of these interactions.

## 2. Methods

### 2.1. Study Setting and Participants

This retrospective cross-sectional study was conducted at an 800-bed tertiary-care teaching hospital in central Thailand. As a medical education center, the facility trains medical students, residents, and fellows while providing comprehensive healthcare services, including specialized departments in cardiology, neurology, oncology, and surgical specialties. The hospital operates multiple intensive care units (ICUs), including medical, surgical, and cardiovascular ICUs. As a tertiary care center, the hospital receives referrals from regional hospitals and offers a range of high-complexity procedures and treatments.

Participants were selected from the electronic health records (EHRs) of patients hospitalized for at least 24 h and subsequently discharged home on warfarin. The recruitment period spanned from 1 January 2019 to 31 December 2022. Patients were included in the cohort if they were 18 years of age or older at discharge and were prescribed warfarin with at least one additional medication concurrently. Only patients discharged from medicine or surgical services were included. Surgical services were treated as a single category. They were not further sub-classified by surgical specialty, although they included general surgery, orthopedic surgery, cardiovascular surgery, and other specialized surgical departments. Medical services included internal medicine, cardiology, pulmonology, and other medical specialties.

For patients with multiple hospitalizations during the recruitment period, only the first admission was included as the index hospitalization, and subsequent admissions for the same patient were excluded to ensure independent observations.

### 2.2. Data Collection

Data were collected from EHRs, pharmacy, and laboratory databases and linked using a unique de-identified code for each patient and admission. The final dataset used for research purposes only was provided by the Information Technology Department of the study hospital. The authors did not have access to information that could identify individual participants during or after data collection. Key variables included patient demographics, discharge services, principal diagnosis, comorbidities, and medication orders.

Warfarin pDDIs were identified using the Micromedex^®^ Drug Interaction database, irrespective of the level of documentation. Micromedex^®^ was selected because it is a widely used, evidence-based drug interaction resource that provides standardized severity ratings and consistent clinical guidance, making it suitable for assessing potential pDDIs in clinical and pharmacoepidemiologic research. Micromedex^®^ classifies interactions by severity as major, moderate, or minor. For this study, we included only pDDIs classified as major severity, defined by Micromedex^®^ as interactions that may be life-threatening or require medical intervention to minimize or prevent serious adverse effects. We included major interactions regardless of the direction of impact on warfarin’s anticoagulant activity. Therefore, our analysis included both types of interactions: those that may enhance warfarin’s effect and those that may attenuate it. As this study examined pDDIs at hospital discharge, we evaluated only medications prescribed concurrently with warfarin that were classified as potential interacting agents in Micromedex^®^. Warfarin dose adjustments and the clinical consequences of these interactions (e.g., changes in INR or bleeding/thrombotic events) were not considered in this cross-sectional analysis.

As a sensitivity analysis to aid clinical interpretation, we re-estimated the prevalence and patient-level burden of major warfarin pDDIs after excluding omeprazole-related interactions, given the context-dependent and often intentional co-prescribing of proton pump inhibitors.

### 2.3. Statistical Analyses

The prevalence of major warfarin pDDIs was estimated for each co-prescribed medication as the proportion of patients receiving the interacting drug among all study participants, expressed as a percentage and 95% CIs. The patient-level burden of major pDDIs was defined as the number of pDDIs identified per individual. To further characterize this burden, the distribution of pDDIs per patient was summarized and stratified by baseline characteristics to highlight variation across patient subgroups. For each stratum, we reported the mean ± standard deviation (SD), median and interquartile range (IQR), and the proportions of patients with ≥1 and ≥2 pDDIs were calculated using the number of patients in each subgroup as the denominator, allowing comparison of burden across subgroups.

We applied log-binomial regression to identify determinants of major warfarin pDDIs, defining the outcome as binary (pDDI presence vs. absence). Log-binomial regression was chosen to estimate risk ratios (RRs), which are more interpretable and avoid the risk inflation associated with odds ratios (ORs) in the context of a highly prevalent outcome.

To assess factors associated with patient-level burden (number of major pDDIs per patient), we fitted Poisson, negative binomial, generalized Poisson, and their zero-inflated variants to account for potential overdispersion, underdispersion, and excess zeros. Model performance was compared using Akaike’s Information Criterion (AIC) and Bayesian Information Criterion (BIC), and the best-fitting model was selected for interpretation. At the same time, consistency of determinants was evaluated across models.

Given the expectation that the major warfarin pDDI burden may increase in a graded manner with the number of medications and to avoid the loss of information from dichotomizing the number of discharge medications into a polypharmacy indicator, we conducted generalized Poisson regression, treating the number of discharge medications as a continuous variable. This allowed a more detailed assessment of the association between the number of medications at discharge and the pDDI burden. Model performance between the binary and continuous specifications of the number of discharge medications was compared using AIC and BIC. Model-based predicted pDDIs and their 95% CIs were subsequently generated using marginal standardization, and the predictions were graphed to illustrate the modeled dose–response relationship.

Collinearity among covariates was assessed using variance inflation factors (VIFs), and covariates with VIFs greater than ten were excluded from the model.

All statistical analyses were performed using Stata Statistical Software: Release 18 (StataCorp, College Station, TX, USA). All hypothesis tests were two-sided, with a significance level of 0.05.

This study is reported in accordance with the Strengthening the Reporting of Observational Studies in Epidemiology (STROBE) guidelines for cross-sectional studies [24] (Supplementary Table S1).

### 2.4. Ethical Considerations

The study was approved by the Human Research Ethics Committee of Thammasat University on 6 April 2023 (approval number: COA 022/2566, research project code: 66PH033). Informed consent was unnecessary because the retrospective data collection posed minimal risk to participants. All data were analyzed anonymously. Data access and handling followed institutional and ethical guidelines to ensure patient confidentiality.

## 3. Results

### 3.1. Study Participants

Table 1 details the baseline characteristics of the 1667 patients at discharge, categorized by the presence of major warfarin pDDIs. Among the cohort, 81.6% of patients were discharged with at least one major pDDI involving warfarin. Patients with pDDIs tended to be older, with a mean age of 64.67 ± 15.02 years, compared with 62.85 ± 16.22 years in those without pDDIs, though this difference was not statistically significant (*p* = 0.058). Significant associations were observed between the presence of major warfarin pDDIs and several baseline characteristics. Patients with pDDIs were more likely to be male (48.9%) compared to those without pDDIs (40.4%) (*p* = 0.007). Additionally, polypharmacy was markedly more common among patients with pDDIs, with 94.6% taking five or more medications at discharge, compared to 75.2% of those without pDDIs (*p* < 0.001).

Additionally, patients discharged from surgical services, as well as those with specific comorbidities such as diabetes mellitus (*p* = 0.015) and hypertension (*p* = 0.009), were more likely to have major warfarin pDDIs. The median LOS was slightly shorter in patients with DDIs (8 days, IQR 4–13) than in those without (9 days, IQR 6–16), with this difference statistically significant (*p* = 0.002). These findings suggest that specific patient populations, particularly those with multiple comorbidities and polypharmacy, are more prone to experiencing the major warfarin pDDIs at hospital discharge.

### 3.2. Prevalence and Burden of the Major Warfarin pDDIs

Table 2 highlights the prevalence of the most common warfarin pDDIs with major severity and the distribution of the number of pDDIs per patient. Omeprazole was the most prevalent interacting drug, implicated in 59.87% (95% CI: 57.47–62.23%) among the patient cohort. Other commonly encountered pDDIs involved aspirin and simvastatin. These medications are routinely prescribed for gastroprotection, antiplatelet therapy, and lipid management, respectively, reflecting their widespread use among patients receiving warfarin. Other notable interacting drugs included clopidogrel, enoxaparin, and amiodarone. Medications with major severity interactions that reduce warfarin’s anticoagulant effect were not excluded from the analysis; however, they did not appear among the most frequently co-prescribed agents in this study cohort. In terms of the number of pDDIs per patient, 1360 (81.6%) experienced at least one major pDDI, with the majority (63.3%) experiencing between one and two major pDDIs. Notably, a minority (18.4%) of patients were without major pDDIs at discharge.

In a sensitivity analysis excluding omeprazole-related interactions, 39.4% of patients had no major warfarin pDDIs at discharge, while 36.7% had one pDDI and 23.9% had two or more pDDIs (Supplementary Table S2).

Table 3 shows the burden of major warfarin pDDIs stratified by patient characteristics. The overall mean number of pDDIs per patient was 1.54 ± 1.16, with a median of 1(IQR 1–2). Nearly four out of five patients (81.6%) had at least one major pDDI, and 46.5% had two or more. Burden was higher in males (mean 1.72 ± 1.18, 51.2% with ≥2 pDDIs) compared with females (mean 1.50 ± 1.15, 45.5% with ≥2 pDDIs). Patients aged ≥65 years had slightly higher burden (mean 1.60 ± 1.16) than those <65 years (1.41 ± 1.14). Burden was also greater among patients discharged from the surgical service (mean 1.71 ± 1.17) than among those discharged from medical wards (mean 1.41 ± 1.13). Polypharmacy showed the strongest association with higher burden (mean 1.63 ± 1.15 vs. 0.58 ± 0.66; ≥2 pDDIs: 50.3% vs. 8.0%)

### 3.3. Determinants of the Major Warfarin pDDIs

Determinants of major warfarin pDDIs are shown in Table 4 and Table 5. The results from Poisson, negative binomial, and generalized Poisson regression models were generally comparable, yielding consistent estimates of the direction and magnitude of the determinants (Table 4). Model fit statistics (AIC and BIC) differed only marginally, suggesting that overdispersion and underdispersion were not substantial in this dataset. Accordingly, the associations between covariates and the number of pDDIs were considered robust across model specifications. Zero-inflated Poisson and zero-inflated negative binomial models were also tested; still, their results and fit indices did not differ meaningfully from the corresponding standard models, implying that excess zeros were not a significant concern in this dataset. Thus, the zero-inflated models were therefore omitted from the final presentation for parsimony.

Log-binomial regression analysis produced consistent results when the outcome was defined as the presence of ≥1 major pDDI (Table 5). Polypharmacy remained the most important predictor (RR 1.72, 95% CI 1.46–2.02; *p* < 0.001). Male sex (RR 1.04, 95% CI 1.00–1.09; *p* = 0.036) and surgical service (RR 1.11, 95% CI 1.06–1.16; *p* < 0.001) were also significantly associated with increased risk of pDDIs. No significant associations were observed for age, comorbidity, or LOS.

**Figure 1:**
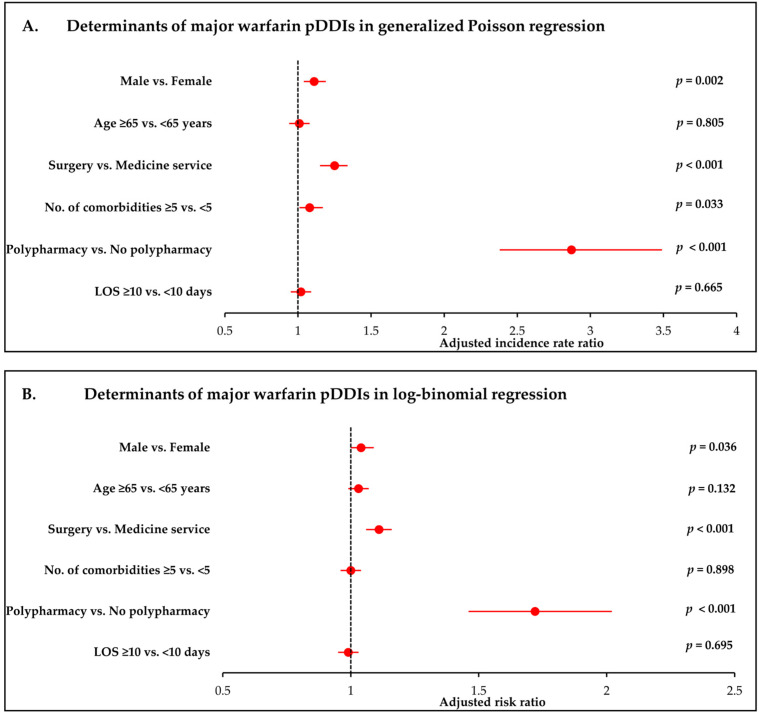
Determinants of major warfarin pDDIs in log-binomial and generalized Poisson regression. (**A**) Adjusted incidence rate ratios (IRRs) for the number of major pDDIs from generalized Poisson regression. (**B**) Adjusted risk ratios (RRs) for the presence of ≥1 major pDDI from log-binomial regression. Both models adjust for age, sex, discharge service, comorbidity burden, polypharmacy, and length of hospital stay. Error bars represent 95% confidence intervals. This figure visually complements the results presented in Table 4 and Table 5.

Figure 1 presents forest plots of the adjusted incidence rate ratios (Panel A) and risk ratios (Panel B) derived from generalized Poisson and log-binomial regression models, respectively. The Figures complement the numerical estimates shown in Table 4 and Table 5 and allow comparison of the magnitude and precision of predictor effects across modelling approaches.

Table 6 presents the results of the generalized Poisson regression model evaluating the association between discharge medication count and the number of major warfarin pDDIs. After adjustment for age, sex, service type, comorbidity burden, and LOS, the number of discharge medications was associated with higher pDDI burden (adjusted IRR 1.09, 95% CI 1.08–1.10). Model fit indices showed lower AIC and BIC values when the number of medications was entered as a continuous variable (AIC 4686, BIC 4729; Table 6) than as a binary variable (AIC 4832, BIC 4876; Table 4), indicating a better model fit with the continuous variable model.

**Figure 2:**
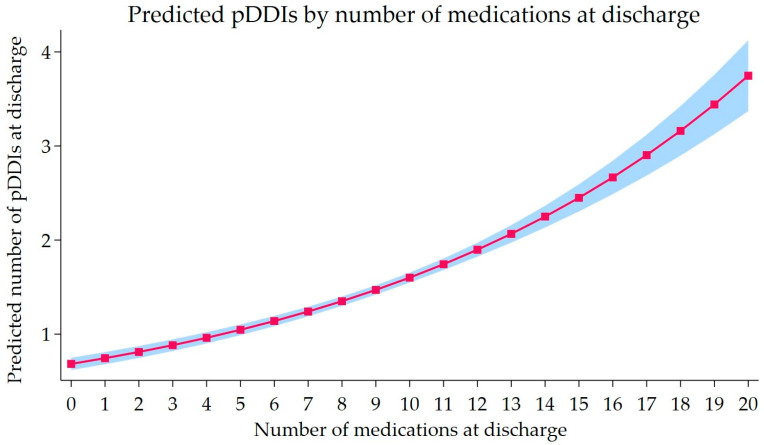
Predicted number of major warfarin pDDIs by number of discharge medications. Predicted pDDI burden from the Generalized Poisson regression, treating the number of discharge medications as a continuous variable. The solid line shows the expected number of major pDDIs for a typical patient, and the shaded band represents the 95% CIs. Predictions are adjusted for age, sex, discharge service, comorbidity burden, and length of stay.

Figure 2 illustrates the corresponding predicted pDDI burdens across the observed range of discharge medication, showing a steadily increasing dose–response pattern. The expected number of major pDDIs increased gradually at lower medication counts and more sharply beyond approximately six medications. The 95% CIs remained narrow throughout the distribution, suggesting stable estimation precision across the observed range. Overall, the model supports a positive dose–response pattern between the number of discharge medications and the major warfarin pDDIs burden.

## 4. Discussion

The present study investigated the prevalence, burden, and determinants of pDDIs at hospital discharge, using warfarin as a model for high-risk medication. Discharge from the hospital has been recognized as a vulnerable period to medication-related problems [2,14]. We observed that more than 80% of patients were discharged with at least one major warfarin pDDI, indicating that potential interactions are common in this setting. Polypharmacy was the most consistent predictor of major pDDIs, and male sex and surgical admissions were also associated with higher burden. While our findings do not provide direct evidence of adverse clinical outcomes, they quantify the exposure to potential interactions at discharge and highlight patient subgroups that would benefit from closer medication review.

Consistent with the high prevalence of warfarin pDDIs found in this study, previous research reported that 54% of older patients with atrial fibrillation experienced warfarin pDDIs at discharge [16]. Another study reported that 35% of serious warfarin pDDIs were identified by pharmacists in post-discharge medication reviews for patients taking warfarin [12]. Although not specifically looking at warfarin pDDIs, Bhandari found that 78.3% of discharge prescriptions had at least one pDDI [11]. Due to the high prevalence of warfarin major pDDIs at discharge and their potential impact on INR, there is a clear need for systematic approaches to manage these interactions during the transition of care. Patients may be at heightened risk for adverse outcomes if their post-discharge INR is nontherapeutic [19,20,21,22]. Implementation of safety measures could include clinical decision support tools to flag potential warfarin pDDIs for providers and enhanced post-discharge follow-up for patients on warfarin therapy.

Across multiple count regression models, polypharmacy consistently emerged as the strongest determinant of major warfarin pDDIs at hospital discharge, demonstrating robustness to model specification. In the log-binomial model, patients with polypharmacy (≥5 medications) were 72% more likely to experience at least one major pDDI. Additionally, the generalized Poisson model showed a nearly threefold increase in the rate of pDDIs per individual. These findings are consistent with prior studies identifying polypharmacy as a key risk factor for pDDIs at discharge among older patients [10]. Importantly, modeling medication count as a continuous variable further demonstrated a graded, dose–response relationship, with each additional discharge medication associated with a statistically significant increase in pDDI burden, without an apparent threshold effect (Figure 2). This cumulative pattern indicates that interaction risk accrues progressively across the full range of medication counts, not only among patients meeting conventional definitions of polypharmacy. This graded pattern reflects a cumulative effect of overall medication burden. It is unlikely to be unique to warfarin, as similar mechanisms may operate for other high-risk medications frequently co-prescribed during care transitions. Accordingly, the continuous modeling approach presented here may provide a generalizable framework for assessing interaction risk among high-risk medications at hospital discharge. From a clinical perspective, these findings underscore the importance of structured medication reconciliation at discharge that considers the overall medication burden, is supported by multidisciplinary review, involves pharmacists for high-risk medications, and, where appropriate, targets deprescribing to mitigate polypharmacy-related interaction risk [25,26,27]. In practice, patients discharged with higher medication counts and those transitioning from surgical services may represent priority groups for structured medication review, pharmacist-led reconciliation, and enhanced discharge planning to reduce cumulative interaction burden.

In the present study, patients discharged from surgical services exhibited a higher risk and burden of major warfarin pDDIs compared with those discharged from medical services. This association was consistent across both count regression and log-binomial models (Table 4 and Table 5), with surgical patients more frequently experiencing multiple concurrent pDDIs (Table 3). Notably, the association between surgical service and pDDI burden persisted after adjustment for medication count, indicating that this finding is not explained solely by polypharmacy. This suggests that factors beyond the number of discharge medications—such as perioperative prescribing patterns and the addition of short-term adjunctive therapies to warfarin regimens—may contribute to the elevated interaction burden observed in surgical patients. Although not specifically focused on warfarin, previous studies in surgical populations have similarly reported higher rates of pDDIs in the postoperative period, underscoring the clinical relevance of medication-related risks during surgical transitions of care [28,29]. By focusing on warfarin as a model high-risk medication, our findings extend these observations and highlight the need for heightened attention to pDDIs among anticoagulated patients discharged from surgical services. Future studies specifically examining service-level prescribing patterns and perioperative medication classes among anticoagulated patients may help clarify the mechanisms underlying this association.

Male sex was independently associated with both the presence and burden of major warfarin pDDIs in adjusted models. Although the effect size was modest, this association was consistent across analyses. The underlying clinical rationale for this finding is uncertain, and sex may serve as a proxy for differences in comorbidity burden, prescribing patterns, or healthcare utilization that were not fully captured in the available data. Accordingly, this finding should be interpreted cautiously, and further studies are needed to determine whether it is consistent across populations or reflects context-specific prescribing practices.

Omeprazole was the most prevalent major warfarin pDDI identified in this study. Pharmacologically, omeprazole weakly inhibits cytochrome P450 (CYP) 2C19 and 3A4; therefore, a significant pharmacokinetic effect is not expected [30,31]. Clinically, however, reported findings are mixed. Observational cohorts reported higher bleeding with warfarin use in combination with proton pump inhibitors (PPIs) versus warfarin alone [32,33], while a large population-based study found lower rates of hospitalization for upper gastrointestinal bleeding with concomitant PPI use [34]. Heterogeneity likely reflects confounding by indication, differing endpoints (e.g., minor vs. major bleeding), exposure definitions, concomitant medications (antiplatelets/non-steroidal anti-inflammatory drugs [NSAIDs]), and anticoagulation quality (e.g., time in therapeutic range). Thus, the clinical impact of PPI co-therapy among warfarin users appears context-dependent and subject to further investigation. Consistent with this interpretation, sensitivity analysis excluding omeprazole-related interactions showed that while the prevalence of any pDDI remained substantial, the proportion of patients with multiple concurrent pDDIs was more markedly reduced. This finding highlights the importance of considering cumulative interaction burden, in addition to overall prevalence, when interpreting database-defined pDDIs.

The high prevalence and burden of major warfarin pDDIs in our study raise important questions about their actual impact on bleeding and thromboembolism. It is important to note that classification of interactions as “major” reflects their potential clinical severity rather than the probability of adverse outcomes. Some interactions, such as those involving antiplatelets or NSAIDs, are well known to increase the risk of bleeding [35]. However, the clinical effects of other frequently identified pDDIs, including PPIs, amiodarone, and several antibiotics, remain heterogeneous and context dependent [30,31,32,33,34,36,37]. Notably, database-defined severity classifications—particularly for commonly flagged interactions such as those involving omeprazole—reflect potential pharmacologic concern rather than a uniform expectation of clinical harm and should therefore be interpreted cautiously in the absence of clinical context.

A large systematic review and meta-analysis by Wang et al. [35], synthesizing evidence from prospective and outcome-based studies, reported increased bleeding risk associated with antiplatelets, NSAIDs, and several antimicrobials, but observed a protective association between PPIs and warfarin-related gastrointestinal bleeding, with no consistent effects on thromboembolic events or mortality across most drug classes. These findings underscore that not all interactions classified as major necessarily translate into clinical harm and highlight the importance of interpreting database-defined pDDI prevalence estimates cautiously. A better understanding of which interactions are clinically consequential—particularly in the context of cumulative pDDI burden at hospital discharge—is essential for improving medication safety for warfarin and other high-risk drugs. Future prospective studies should evaluate whether higher discharge pDDI burden—particularly multiple concurrent major interactions—is associated with anticoagulation instability (e.g., INR variability) and downstream bleeding or thromboembolic outcomes, and whether targeted discharge-focused interventions can mitigate these risks.

Several limitations should be acknowledged. First, the observational design introduces the possibility of residual confounding, as some relevant clinical or prescribing factors may not have been captured. Second, only selected covariates were included in the regression models to reduce collinearity and overfitting, potentially limiting the determinants assessed. Third, the analysis focused on pDDIs rather than on observed adverse clinical outcomes; therefore, the clinical significance of these interactions cannot be inferred directly. Accordingly, the high prevalence of database-defined major pDDIs observed in this study should not be interpreted as a direct estimate of patient harm, but rather as an indication of potential exposure requiring cautious clinical interpretation. Finally, as the study population was drawn from a tertiary care center in Thailand, the findings may not be generalizable to other settings or populations. The generalizability of these findings to other healthcare settings or broader populations requires further investigation. Nevertheless, the observed association between increasing medication burden and cumulative pDDI risk is consistent with pharmacologic principles and prior studies, suggesting that the relationship between polypharmacy and interaction burden is likely relevant across healthcare systems. In addition, the consistency of associations across different modeling approaches provides reassurance regarding the robustness of the identified determinants, particularly polypharmacy.

## 5. Conclusions

Using warfarin as a model high-risk medication, we found that major pDDIs were highly prevalent at hospital discharge in a tertiary-care hospital in Thailand. Across both log-binomial and generalized Poisson models, polypharmacy was consistently associated with a higher likelihood and greater burden of major pDDIs. These findings highlight the importance of careful medication review and consideration of overall medication burden during care transitions for patients receiving high-risk medications.

## Figures and Tables

**Table 1:** Patient characteristics at discharge (*N* = 1667).

Presence of Warfarin pDDIs with Major Severity			
	No pDDIs *n* (%)	Yes (≥1 pDDI) *n* (%)	*p*-Value *
Total	307 (18.4%)	1360 (81.6%)	-
Age in years (mean ± SD)	62.85 ± 16.22	64.67 ± 15.02	0.058
Age ≥ 65 years	109 (48.2%)	518 (55.2%)	0.026
Male	124 (40.4%)	665 (48.9%)	0.007
Polypharmacy (≥5 medications at discharge)	231 (75.2)	1286 (94.6%)	<0.001
LOS in days (median, IQR)	9 (6 –16)	8 (4–13)	0.002
LOS ≥ 10 days	133 (43.3%)	654 (48.1%)	0.131
Discharge service			<0.001
Medicine	208 (67.8%)	746 (54.9%)	
Surgery	99 (32.2%)	614 (45.1%)	
Number of comorbidities (median, IQR)	4 (3–6)	5 (3–6)	0.730
Number of comorbidities ≥ 5	154 (50.2%)	673 (49.5%)	0.830
Comorbidities			
Atrial fibrillation	149 (48.5%)	544 (40.0%)	0.006
Chronic heart failure	53 (17.3%)	198 (14.6%)	0.231
Chronic kidney disease	39 (12.7%)	195 (14.3%)	0.456
Coronary artery disease	21 (6.8%)	295 (21.7%)	<0.001
Dyslipidemia	76 (24.8%)	357 (26.2%)	0.590
Diabetes mellitus	61 (19.9%)	361 (26.5%)	0.015
Hypertension	128 (41.7%)	680 (50.0%)	0.009
Ischemic stroke	30 (9.8%)	154 (11.3%)	0.433
Mitral valve stenosis	40 (13.0%)	74 (5.4%)	<0.001
Replacement of mechanical mitral valve	11 (3.6%)	49 (3.6%)	0.987
Replacement of mechanical aortic valve	7 (2.3%)	57 (4.2%)	0.115
Venous thromboembolism	6 (2.0%)	24 (1.8%)	0.821

* Independent *t*-test for quantitative variables, Chi-square test for categorical variables, and Wilcoxon rank-sum test for the difference between the medians. Abbreviations: pDDIs, potential drug–drug interactions; IQR, interquartile range; LOS, length of stay.

**Table 2:** Prevalence and burden of major warfarin pDDIs at hospital discharge (*N* = 1667).

(a) The 10 Most Frequent Interacting Drugs		
Interacting Drugs	*n*	% (95% CIs)
Omeprazole	998	59.87 (57.47–62.23)
Aspirin	378	22.68 (20.68–24.76)
Simvastatin	243	14.58 (12.91–16.36)
Clopidogrel	151	9.06 (7.72–10.54)
Enoxaparin	137	8.22 (6.94–9.64)
Amiodarone	88	5.28 (4.26–6.46)
Amoxicillin/clavulanate	87	5.22 (4.20–6.40)
Allopurinol	74	4.44 (3.50–5.54)
Cephalexin	44	2.64 (1.92–3.53)
Cefdinir	39	2.34 (1.67–3.18)
**(b) Patient-Level Burden of pDDIs**		
**Number of pDDIs per Patient**	***n***	**% (95% CIs)**
0	307	18.42 (16.58–20.36)
1	585	35.09 (32.80–37.44)
2	471	28.25 (26.10–30.48)
3	218	13.08 (11.49–14.79)
4	61	3.66 (2.81–4.68)
≥5	25	1.50 (0.97–2.21)

**Table 3:** Burden of major warfarin pDDIs per patient, stratified by patient characteristics.

Patient Characteristics	*n*	Mean ± SD	Median (IQR)	≥1 pDDI *n* (%)	≥2 pDDIs *n* (%)
Overall	1667	1.54 ± 1.16	1 (1–2)	1360 (81.58)	775 (46.49)
Gender					
Female	1386	1.50 ± 1.15	1 (1–2)	1113 (80.30)	631 (45.53)
Male	281	1.72 ± 1.18	2 (1–2)	247 (87.90)	144 (51.25)
Age					
<65 years	556	1.41 ± 1.14	1 (1–2)	433 (77.88)	230 (41.37)
≥65 years	1111	1.60 ± 1.16	1 (1–2)	927 (83.44)	545 (49.05)
Service					
Medicine	954	1.41 ± 1.13	1 (1–2)	746 (78.20)	393 (41.19)
Surgery	713	1.71 ± 1.17	2 (1–2)	614 (86.12)	382 (53.58)
No. of comorbidities					
<5	840	1.49 ± 1.10	1 (1–2)	687 (81.79)	377 (44.88)
≥5	827	1.59 ± 1.21	1 (1–2)	673 (81.38)	398 (48.13)
Polypharmacy					
No	150	0.58 ± 0.66	0 (0–1)	74 (49.33)	12 (8.00)
Yes	1517	1.63 ± 1.15	2 (1–2)	1286 (84.77)	763 (50.30)
LOS					
<10 days	880	1.46 ± 1.13	1 (1–2)	706 (80.23)	383(43.52)
≥10 days	787	1.62 ± 1.18	1 (1–2)	654 (83.10)	392(49.81)

Abbreviations: pDDIs, potential drug–drug interactions; LOS, length of stay.

**Table 4:** Determinants of major warfarin pDDIs in count regression models (*N* = 1667).

Determinants	Adjusted Incidence Rate Ratio (95% CI), *p*-Value		
	Poisson	Negative Binomial	Generalized Poisson
Male (Reference: female)	1.11 (1.04–1.19), *p* = 0.003	1.11 (1.04–1.19), *p* = 0.003	1.11 (1.04–1.19), *p* = 0.002
Age ≥ 65 years (Reference: age < 65 years)	1.02 (0.95–1.09), *p* = 0.627	1.02 (0.95–1.09), *p* = 0.627	1.01 (0.94–1.08), *p* = 0.850
Surgery service (Reference: medicine service)	1.25 (1.16–1.35), *p* < 0.001	1.25 (1.16–1.35), *p* < 0.001	1.24 (1.15–1.34), *p* < 0.001
No. of comorbidities ≥ 5 (Reference: no. of comorbidities < 5)	1.08 (1.00–1.16), *p* = 0.049	1.08 (1.00–1.16), *p* = 0.049	1.08 (1.01–1.17), *p* = 0.033
Polypharmacy (Reference: no polypharmacy)	2.78 (2.31–3.35), *p* < 0.001	2.78 (2.31–3.34), *p* < 0.001	2.87 (2.36–3.49), *p* < 0.001
LOS ≥ 10 days (Reference: LOS < 10 days)	1.02 (0.95–1.09), *p* = 0.606	1.02 (0.95–1.09), *p* = 0.606	1.02 (0.95–1.09), *p* = 0.665
AIC	4872	4872	4832
BIC	4910	4910	4876

Note: Results from Poisson and negative binomial models are presented for comparison. The generalized Poisson model was selected as the primary model based on model fit and is the focus of interpretation. Abbreviations: AIC, Akaike’s Information Criteria; BIC, Bayesian Information Criteria; pDDIs, potential drug–drug interactions; LOS, length of stay.

**Table 5:** Determinants of major warfarin pDDIs in multivariable log-binomial regression (*N* = 1667).

Determinants	Log-Binomial Model		
	Adjusted RR	95% CIs	*p*-Value
Male (Reference: female)	1.04	1.00–1.09	0.036
Age ≥ 65 years (Reference: age < 65 years)	1.03	0.99–1.07	0.132
Surgery service (Reference: medicine service)	1.11	1.06–1.16	<0.001
No. of comorbidities ≥ 5 (Reference: no. of comorbidities < 5)	1.00	0.96–1.04	0.898
Polypharmacy (Reference: no polypharmacy)	1.72	1.46–2.02	<0.001
LOS ≥ 10 days (Reference: LOS < 10 days)	0.99	0.95–1.03	0.695

Note: This Table presents determinants of the presence of ≥1 major warfarin pDDI at discharge. Abbreviations: pDDIs, potential drug–drug interactions; LOS, length of stay; RR, risk ratio.

**Table 6:** Association between the number of discharge medications and the burden of major warfarin pDDIs at hospital discharge using generalized Poisson regression (*N* = 1667).

Determinants	Generalized Poisson Regression		
	Adjusted IRR	95% CIs	*p*-Value
Male (Reference: female)	1.12	1.03–1.21	0.009
Age ≥ 65 years (Reference: age < 65 years)	0.96	0.90–1.03	0.315
Surgery service (Reference: medicine service)	1.21	1.13–1.30	<0.001
No. of comorbidities ≥ 5 (Reference: no. of comorbidities < 5)	0.95	0.88–1.03	0.197
LOS ≥ 10 days (Reference: LOS < 10 days)	0.93	0.87–0.99	0.039
Number of discharge medications *	1.09	1.08–1.10	<0.001

The model fit: Dispersion = −0.1365051; Log pseudolikelihood = −2334.8749; Likelihood-ratio χ^2^(6) = 341.08, *p* < 0.000; AIC 4686; BIC 4729. * The number of discharge medications was modeled as a continuous variable. Note: This Table presents a focused analysis examining the association between the number of discharge medications at discharge and the burden of major warfarin pDDIs. Abbreviations: pDDIs, potential drug–drug interactions; LOS, length of stay; IRR, incidence rate ratio.

## Data Availability

The data presented in this study are available upon reasonable request from the corresponding author. The data are not publicly available due to privacy or ethical restrictions.

## Supplementary Materials

The following supporting information can be downloaded at: https://www.mdpi.com/article/10.3390/clinpract16010008/s1, Table S1: STROBE Statement—Checklist of items that should be included in reports of cross-sectional studies; Table S2: Sensitivity analysis of the prevalence and burden of major warfarin pDDIs at hospital discharge excluding omeprazole-related interactions (*N* = 1667).
