# Supplementary material for: Burden and Determinants of Drug–Drug Interactions at Hospital Discharge: Warfarin as a Model for High-Risk Medication Safety
Source: Clin Pract. 2025 Dec 31;16(1):8. doi: 10.3390/clinpract16010008 (PMC12839695; doi:10.3390/clinpract16010008)
Supplement: Supplementary file 1 [file clinpract-16-00008-s001.zip › clinpract-4025688-supplementary.pdf]

clinics and practice

MDPI

Supplementary Table S1. STROBE Statement—Checklist of items that should be included in reports of cross-sectional studies

	Item No	Recommendation	Yes/No/NA	Comments
**Title and abstract**	1	(*a*) Indicate the study's design with a commonly used term in the title or the abstract	Yes	Abstract, Methods subsection.
		(*b*) Provide in the abstract an informative and balanced summary of what was done and what was found	Yes	Abstract, Results subsection.
**Introduction**				
Background/rationale	2	Explain the scientific background and rationale for the investigation being reported	Yes	Introduction, paragraphs 1 to 3.
Objectives	3	State specific objectives, including any prespecified hypotheses	Yes	Introduction, final paragraph.
**Methods**				
Study design	4	Present key elements of study design early in the paper	Yes	Methods section 2.1 (study setting and participants).
Setting	5	Describe the setting, locations, and relevant dates, including periods of recruitment, exposure, follow-up, and data collection	Yes	Methods section 2.1 (study setting and participants).
Participants	6	(*a*) Give the eligibility criteria, and the sources and methods of selection of participants	Yes	Methods section 2.1 (study setting and participants).
Variables	7	Clearly define all outcomes, exposures, predictors, potential confounders, and effect modifiers. Give diagnostic criteria, if applicable	Yes	Methods section 2.2 (data collection).
Data sources/ measurement	8	For each variable of interest, give sources of data and details of methods of assessment (measurement). Describe comparability of assessment methods if there is more than one group	Yes	Methods section 2.2 (data collection).
Bias	9	Describe any efforts to address potential sources of bias	Yes	Methods sections 2.1, 2.2, and 2.4; Discussion. Potential selection, misclassification, and confounding biases were addressed through predefined

Supplementary Table S1. STROBE Statement—Checklist of items that should be included in reports of cross-sectional studies

	Item No	Recommendation	Yes/No/NA	Comments
				inclusion criteria, use of index admissions, multivariable adjustment, sensitivity analyses, and cautious interpretation of database-defined pDDIs.
Study size	10	Explain how the study size was arrived at	NA	Because this was a retrospective cross-sectional study, all eligible patients meeting the inclusion criteria during the study period were included, and no formal sample size calculation was performed.
Quantitative variables	11	Explain how quantitative variables were handled in the analyses. If applicable, describe which groupings were chosen and why	Yes	Methods section 2.3 (statistical analyses). Quantitative variables were analyzed as continuous or categorical as appropriate. Length of stay and number of comorbidities were categorized using median values for descriptive stratification due to skewed distributions. The number of discharge medications was modeled both categorically (polypharmacy ≥5 medications) and continuously to assess graded associations.
Statistical methods	12	(*a*) Describe all statistical methods, including those used to control for confounding	Yes	Methods section 2.3 (statistical analyses).
		(*b*) Describe any methods used to examine subgroups and interactions	NA	There were no planned subgroup or interaction analyses in this study.
		(*c*) Explain how missing data were addressed	NA	There was no missing data in the present study.
		(*d*) If applicable, describe analytical methods taking account of sampling strategy	NA	There was no planned sampling strategy in the present study.
		(*e*) Describe any sensitivity analyses	Yes	Methods section 2.3 (statistical analyses).
**Results**				
Participants	13	(a) Report numbers of individuals at each stage of study—eg numbers potentially eligible, examined for eligibility, confirmed eligible, included in the study,	Yes	All eligible patients meeting the inclusion criteria during the study period were included; the final analytic sample consisted of 1,667 participants.

https://doi.org/10.3390/clinpract16010008

Supplementary Table S1. STROBE Statement—Checklist of items that should be included in reports of cross-sectional studies

	Item No	Recommendation	Yes/No/NA	Comments
		completing follow-up, and analysed		
		(b) Give reasons for non-participation at each stage	NA	This was a retrospective cross-sectional study using electronic health records.
		(c) Consider use of a flow diagram	NA	All eligible participants were included in the study.
Descriptive data	14	(a) Give characteristics of study participants (eg demographic, clinical, social) and information on exposures and potential confounders	Yes	Results section 3.1 (study participants).
		(b) Indicate number of participants with missing data for each variable of interest	NA	There was no missing data in the present study.
Outcome data	15	Report numbers of outcome events or summary measures	Yes	Results section 3.2 (prevalence and burden of the major warfarin pDDIs).
Main results	16	(*a*) Give unadjusted estimates and, if applicable, confounder-adjusted estimates and their precision (eg, 95% confidence interval). Make clear which confounders were adjusted for and why they were included	Yes	Results section 3.2 (prevalence and burden of the major warfarin pDDIs), and 3.3 (determinants of the major warfarin pDDIs).
		(*b*) Report category boundaries when continuous variables were categorized	Yes	Results section 3.1 (study participants).
		(*c*) If relevant, consider translating estimates of relative risk into absolute risk for a meaningful time period	NA	Because this was not an interventional study.
Other analyses	17	Report other analyses done—eg analyses of subgroups and interactions, and sensitivity analyses	Yes	Results section 3.2 (prevalence and burden of the major warfarin pDDIs). Sensitivity analysis excluding omeprazole-related interactions on prevalence and burden of major warfarin pDDIs was reported and shown in Supplementary Table S2.
**Discussion**				
Key results	18	Summarise key results with reference to study objectives	Yes	Within Discussion.

https://doi.org/10.3390/clinpract16010008

Supplementary Table S1. STROBE Statement—Checklist of items that should be included in reports of cross-sectional studies

	Item No	Recommendation	Yes/No/NA	Comments
Limitations	19	Discuss limitations of the study, taking into account sources of potential bias or imprecision. Discuss both direction and magnitude of any potential bias	Yes	Within Discussion.
Interpretation	20	Give a cautious overall interpretation of results considering objectives, limitations, multiplicity of analyses, results from similar studies, and other relevant evidence	Yes	Within Discussion.
Generalisability	21	Discuss the generalisability (external validity) of the study results	Yes	Within Discussion. Generalizability should be cautious given the data from a single center in Thailand; however, the results are consistent with previous studies in other settings.
**Other information**				
Funding	22	Give the source of funding and the role of the funders for the present study and, if applicable, for the original study on which the present article is based	NA	The present study received no external funding.

NA, non-applicable.

Supplementary Table S2. Sensitivity analysis of the prevalence and burden of major warfarin pDDIs at hospital discharge excluding omeprazole-related interactions (*N* = 1667)

(a) The 10 most frequent interacting drugs		
**Interacting drugs**	*n*	**% (95% CIs)**
Aspirin	378	22.68 (20.68–24.76)
Simvastatin	243	14.58 (12.91–16.36)
Clopidogrel	151	9.06 (7.72–10.54)
Enoxaparin	137	8.22 (6.94–9.64)
Amiodarone	88	5.28 (4.26–6.46)
Amoxicillin/clavulanate	87	5.22 (4.20–6.40)
Allopurinol	74	4.44 (3.50–5.54)
Cephalexin	44	2.64 (1.92–3.53)
Cefdinir	39	2.34 (1.67 – 3.18)
Ciprofloxacin	28	1.68 (1.12 – 2.42)
**(b) Patient-level burden of pDDIs**		
**Number of pDDIs per patient**	*n*	**% (95% CIs)**
0	657	39.41 (37.06–41.80)
1	611	36.65 (34.33–39.02)
2	288	17.28 (15.49–19.18)
3	80	4.80 (3.82–5.94)
4	23	1.38 (0.88–2.06)
≥5	8	0.48 (0.21–0.94)

Disclaimer/Publisher's Note: The statements, opinions and data contained in all publications are solely those of the individual author(s) and contributor(s) and not of MDPI and/or the editor(s). MDPI and/or the editor(s) disclaim responsibility for any injury to people or property resulting from any ideas, methods, instructions or products referred to in the content.

https://doi.org/10.3390/clinpract16010008
